# Managing, Analysing, and Integrating Big Data in Medical Bioinformatics: Open Problems and Future Perspectives

**DOI:** 10.1155/2014/134023

**Published:** 2014-09-01

**Authors:** Ivan Merelli, Horacio Pérez-Sánchez, Sandra Gesing, Daniele D'Agostino

**Affiliations:** ^1^Bioinformatics Research Unit, Institute for Biomedical Technologies, National Research Council of Italy, Segrate, 20090 Milan, Italy; ^2^Bioinformatics and High Performance Computing Research Group (BIO-HPC), Computer Science Department, Universidad Católica San Antonio de Murcia (UCAM), 30107 Murcia, Spain; ^3^Department of Computer Science and Engineering, Center for Research Computing, University of Notre Dame, P.O. Box 539, Notre Dame, IN 46556, USA; ^4^Advanced Computing Systems and High Performance Computing Group, Institute of Applied Mathematics and Information Technologies, National Research Council of Italy, 16149 Genoa, Italy

## Abstract

The explosion of the data both in the biomedical research and in the healthcare systems demands urgent solutions. In particular, the research in omics sciences is moving from a hypothesis-driven to a data-driven approach. Healthcare is additionally always asking for a tighter integration with biomedical data in order to promote personalized medicine and to provide better treatments. Efficient analysis and interpretation of Big Data opens new avenues to explore molecular biology, new questions to ask about physiological and pathological states, and new ways to answer these open issues. Such analyses lead to better understanding of diseases and development of better and personalized diagnostics and therapeutics. However, such progresses are directly related to the availability of new solutions to deal with this huge amount of information. New paradigms are needed to store and access data, for its annotation and integration and finally for inferring knowledge and making it available to researchers. Bioinformatics can be viewed as the “glue” for all these processes. A clear awareness of present high performance computing (HPC) solutions in bioinformatics, Big Data analysis paradigms for computational biology, and the issues that are still open in the biomedical and healthcare fields represent the starting point to win this challenge.

## 1. Introduction

The increasing availability of omics data resulting from improvements in the acquisition of molecular biology results and in systems biology simulation technologies represents an unprecedented opportunity for bioinformatics researchers, but also a major challenge. A similar scenario arises for the healthcare systems, where the digitalization of all clinical exams and medical records is becoming a standard in hospitals. Such huge and heterogeneous amount of digital information, nowadays called Big Data, is the basis for uncovering hidden patterns in data, since it allows the creation of predictive models for real-life biomedical applications. But the main issue is the need of improved technological solutions to deal with them.

A simple definition of Big Data is based on the concept of data sets whose size is beyond the management capabilities of typical relational database software. A more articulated definition of Big Data is based on the three versus paradigm:* volume*,* variety,* and* velocity* [1]. The volume recalls for novel storage scalability techniques and distributed approaches for information query and retrieval. The second V, the variety of the data source, prevents the straightforward use of neat relational structures. Finally, the increasing rate at which data is generated, the velocity, has followed a similar pattern as the volume. This “need for speed,” particularly for web-related applications, has driven the development of techniques based on key-value stores and columnar databases behind portals and user interfaces, because they can be optimized for the fast retrieval of precomputed information. Thus, smart integration technologies are required for merging heterogeneous resources: promising approaches are the use of technologies relying on lighter placement with respect to relational databases (i.e., NoSQL databases) and the exploitation of semantic and ontological annotations.

Although the Big Data definition can still be considered quite nebulous, it does not represent just a keyword for researchers or an abstract problem: the USA Administration launched a 200 million dollar “Big Data Research and Development Initiative” in March 2012, with the aim to improve tools and techniques for the proper organization, efficient access, and smart analysis of the huge volume of digital data [2]. Such a high amount of investments is justified by the benefit that is expected from processing the data, and this is particularly true for omics science. A meaningful example is represented by the projects for population sequencing. The first one is the 1000 genomes [3], which provides researchers with an incredible amount of raw data. Then, the ENCODE project [4], a follow-up to the Human Genome Project (Genomic Research) [5], is having the aim of identifying all functional elements in the human genome. Presently, this research is moving at a larger scale: as clearly appears considering the Genome 10K project [6] and the more recent 100K Genomes Project [7]. Just to provide an order of magnitude, the amount of data produced in the context of the 1000 Genomes Project is estimated in 100 Terabytes (TB), and the 100K Genomes Project is likely to produce 100 times such data. The targeting cost for sequencing a single individual will reach soon $1000 [8], which is affordable not only for large research projects but also for individuals. We are running into the paradox that the cheapest solution to cope with these data will be to resequence genomes when analyses are needed instead of storing them for future reuse [9].

Storage represents only one side of the medal. The final goal of research activities in omics sciences is to turn such amount of data into usable information and real knowledge. Biological systems are very complex, and consequently the algorithms involved in analysing them are very complex as well. They still require a lot of effort in order to improve their predictive and analytical capabilities. The real challenge is represented by the automatic annotation and/or integration of biological data in real-time, since the objective to reach is to understand them and to achieve the most important goal in bioinformatics: mining information.

**Figure 1:**
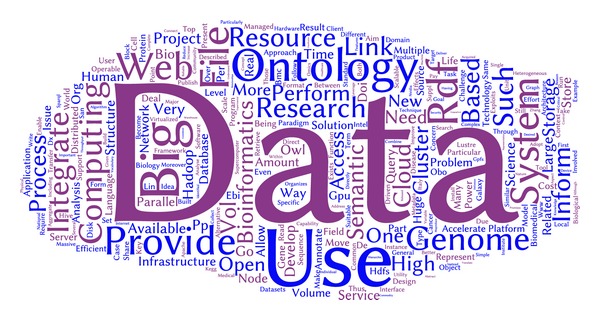
Tag Crowd of the concepts presented in the paper.

In this work we present a brief review of the technological aspects related to Big Data analysis in biomedical informatics. The paper is structured as follows. In Section 2 architectural solutions for Big Data are described, paying particular attention to the needs of the bioinformatics community.  Section 3 presents parallel platforms for Big Data elaboration, while Section 4 is concerned with the approaches for data annotation, specifically considering the methods employed in the computational biology field.  Section 5 introduces data access measures and security for biomedical data. Finally, Section 6 presents some conclusions and future perspective. A Tag Crowd diagram of the concepts presented in the paper is shown in Figure 1.

## 2. Big Data Architectures

Domains concerned with data-intensive applications have in common the abovementioned three versus, even though the actual way by which this information is acquired, stored, and analysed can vary a lot from field to field. The main common aspect is represented by the requirements for the underlying IT architecture. The mere availability of disk arrays of several hundreds of TBs is in fact not sufficient, because the access to the data will have, statistically, some fails [10]. Thus, reliable storage infrastructures have to be robust with respect to these problems. Moreover, the analysis of Big Data needs frequent data access for the analysis and integration of information, resulting in considerable data transfer operations. Even though we can assume the presence of a sufficient amount of bandwidth inside a cluster, the use of distributed computing infrastructure requires adopting effective solutions. Other aspects have also to be addressed, as secure access policies to both the raw data and the derived results. Choosing a specific architecture and building an appropriate Big Data system are challenging because of diverse heterogeneous factors. All the major vendors as IBM [11], Oracle [12], and Microsoft [13] propose solutions (mostly business-oriented) based on their software ecosystems. Here we will discuss the major architectural aspects taking into account open source projects and scientific experiences.

### 2.1. Managing and Accessing Big Data

The first and obvious concern with Big Data is the volume of information that researchers have to face, especially in bioinformatics. At lower level this is an IT problem of file systems and storage reliability, whose solution is not obvious and not unique. Open questions are what file system to choose and will the network be fast enough to access this data. The issue arising in data access and retrieval can be highlighted with a simple consideration [14]: scanning data on a modern physical hard disk can be done with a throughput of about 100 Megabytes/s. Therefore, scanning 1 Terabyte takes 5 hours and 1 Petabyte takes 5000 hours. The Big Data problem does not only rely in archiving and conserving huge quantity of data. The real challenge is to access such data in an efficient way, applying massive parallelism not only for the computation, but also for the storage.

Moreover, the transfer rate is inversely proportional to the distance to cover. HPC clusters are typically equipped with high-level interconnections as InfiniBand, which have a latency of 2000 ns (only 20 times slower than RAM and 200 times faster than a Solid State Disk) and data rates ranging from 2.5 Gigabit per second (Gbps) with a single data rate link (SDR), up to 300 Gbps with a 12-link enhanced data rate (EDR) connection. But this performance can only be achieved on a LAN, and the real problems arise when it is necessary to transfer data between geographically distributed sites, because the Internet connection might not suitable to transfer Big Data. Although several projects, such GÉANT [15], the pan-European research and education network, and its US counterpart Internet2 [16], have been funded to improve the network interconnections among states; the achievable bandwidth is insufficient. For example, BGI, the world largest genomics service provider, uses FedEx for delivering results [17].

If a local infrastructure is exploited for the analysis of Big Data, one effective solution is represented by the use of client/server architectures where the data storage is spread among several devices and made accessible through a (local) network. The available tools can be mainly subdivided in* distributed file systems*,* cluster file systems*, and* parallel file systems*. Distributed file systems consist of disk arrays physically attached to a few I/O servers through fast networks and then shared to the other nodes of a cluster. In contrast,* cluster file systems* provide direct disk access from multiple computers at the block level (access control must take place on the client node).* Parallel file systems* are like* cluster file systems*, where multiple processes are able to concurrently read and write a single file, but exploit a client-server approach, without direct access to the block level. An exhaustive taxonomy and a survey are provided in [18].

Two of the highest performance parallel file systems are the General Parallel File System (GPFS) [19], developed by IBM, and Lustre [20], an open source solution. Most of the supercomputers employ them: in particular, Lustre is used in Titan, the second supercomputer of the TOP500 list (November 2013) [21]. The Titan storage subsystem contains 20,000 disks, resulting in 40 Petabyte of storage and about 1 Terabyte/sec of storage bandwidth. As regards GPFS, it was recently exploited within the Elastic Storage [22], the IBM file management solution working as a control plane for smart data handling. The software can automatically move less frequently accessed data to the less expensive storage available in the infrastructure, while leaving faster and more expensive storage resources (i.e., SSD disk or flash) for more important data. The management is guided by analytics, using patterns, storage characteristics, and the network to determine where to move the data.

A different solution is represented by the Hadoop Distributed File System (HDFS) [23], a Java-based file system that provides scalable and reliable data storage that is designed to span large clusters of commodity servers. It is an open source version of the GoogleFS [24] introduced in 2003. The design principles were derived from Google's needs, as the fact that most files only grow because new data have to be appended, rather than overwriting the whole file, and that high sustained bandwidth is more important than low latency. As regards I/O operations, the key aspects are the efficient support for large streaming or small random reads, besides large and sequential writes to append data to files. The other operations are supported as well, but they can be implemented in a less efficient way.

At the architectural level, HDFS requires two processes: a* NameNode* service, running on one node in the cluster and a* DataNode* process running on each node that will process data. HDFS is designed to be fault-tolerant due to replication and distribution of data, since every loaded file is replicated (3 times is the default value) and split into blocks that are distributed across the nodes. The* NameNode* is responsible for the storage and management of metadata, so that when an application requires a file, the* NameNode* informs about the location of the needed data. Whenever a data node is down, the* NameNode* can redirect the request to one of the replicas until the data node is back online. Since the cluster size can be very large (it was demonstrated with clusters up to 4,000 nodes), the single* NameNode* per cluster forms a possible bottleneck and single point of failure. This can be mitigated by the fact that metadata can be stored in the main memory and the recent HDFS High Availability feature provides the user with the option of running two redundant* NameNodes* in the same cluster, one of them in standby, but ready to intervene in case of failure of the other.

### 2.2. Middleware for Big Data

The file system is the first level of the architecture. The second level corresponds to the framework/middleware supporting the development of user-specific solutions, while applications represent the third level. Besides the general-purpose solutions for parallel computing like the Message Passing Interface [25], hybrid solutions based on it [26, 27], or extension to specific frameworks like the R software environment for statistical computing [28], there are several tools specifically developed for Big Data analysis.

The first and more famous example is the abovementioned Apache Hadoop [23], an open source software framework for large-scale storing and processing of data sets on distributed commodity hardware. Hadoop is composed of two main components, HDFS and MapReduce. The latter is a simple framework for distributed processing based on the* Map* and* Reduce* functions, commonly used in functional programming. In the* Map* step the input is partitioned by a master process into smaller subproblems and then distributed to worker processes. In the* Reduce* step the master process collects the results and combines them in some way to provide the answer to the problem it was originally trying to solve.

Hadoop was designed as a platform for an entire ecosystem of capabilities [29], used in particular by a large number of companies, vendors, and institutions [30]. To provide an example in the bioinformatics field, in The Cancer Genome Atlas project researchers implemented the process of “sharding,” splitting genome data into smaller more manageable chunks for cluster-based processing, utilising the Hadoop framework and the Genome Analysis Toolkit (GATK) [31, 32]. Many other works based on Hadoop are present in literature [33–36]; for example, some specific libraries were developed as Hadoop-BAM, a library for distributed processing of genetic data from next generation sequencer machines [37], and Seal, a suite of distributed applications for aligning short DNA reads, manipulating short read alignments, and analyse the achieved results [38], was also developed relying on this framework.

Hadoop was the basis for other higher-level solutions as Apache Hive [39], a distributed data warehouse infrastructure for providing data summarization, query, and analysis. Apache Hive supports analysis of large datasets stored in HDFS and compatible file systems such as the Amazon S3 file system. It provides an SQL-like language called HiveQL while maintaining full support for map/reduce. To accelerate queries, it provides indexes, including bitmap indexes, and it is worth exploiting in several bioinformatics applications [40]. Apache Pig [41] has the similar aim to allow domain experts, who are not necessarily IT specialists, to write complex MapReduce transformations using a simpler scripting language. It was used for example for sequence analysis [33].

Hadoop is considered almost as synonymous for Big Data. However, there are some alternatives based on the same MapReduce paradigm like Disco [42], a distributed computing framework aimed at providing a MapReduce platform for Big Data processing using Python application, that can be coupled with the Biopython toolkit [43] of the Open Bioinformatics Foundation, Storm [44], a distributed real-time computation system for processing fast, and large streams of data and proprietary systems, for example, from Software AG, LexisNexis, and ParStream (see their websites for more information).

## 3. Computational Facilities for Analysing Big Data

The traditional platforms for operating the software frameworks that facilitate Big Data analysis are HPC clusters, possibly accessed via grid computing infrastructures [45]. Such approach has however the possible drawback to provide an insufficient possibility to customize the computational environment if the computing facilities are not owned by the scientists that will analyze the data. This is one of the reasons why cloud computing services increased their importance as economic solutions to perform large-scale analysis on an as-needed basis, in particular for medium-small laboratories that cannot afford the cost to buy and maintain a sufficiently powerful infrastructure [46]. In this section we will very briefly review bioinformatics applications or projects exploiting these platforms.

### 3.1. Cluster Computing

The data parallel approach, that is, the parallelization paradigm that subdivides the data to analyse among almost independent processes, is a suitable solution for many kinds of Big Data analysis that results in high scalability and performance figures. The key issues while developing applications using data parallelism are the choice of the algorithm, the strategy for data decomposition, load balancing among possibly heterogeneous computing nodes, and the overall accuracy of the results [47].

An example of Big Data analysis based on this approach in the field of bioinformatics concerns the* de novo* assembly algorithms, which typically work finding the fragments that “overlap” in the sequenced reads and recording these overlaps in a huge diagram called de Bruijn (or assembly) graph [48]. For a large genome, this graph can occupy many Terabytes of RAM, and completing the genome sequence can require days of computation on a world-class supercomputer. This is the reason why memory distributed approaches, such as Abyss [49], are now widely exploited, although the implementation of efficient multisever approaches has required huge effort and is still under active development.

Generally, we can say that data parallel approaches are straightforward solutions for inferring correlations, but not for causality. In these cases semantics and ontological techniques are of particular importance, as those described in Section 4.

### 3.2. GPU Computing

HPC technologies are the forefront of accelerated data analysis revolutions, making it possible to carry out processing breakthroughs that would directly translate into real benefits for the society and the environment. The use of accelerator devices such as GPUs represents a cost-effective solution able to support up to 11.5 Teraflops within a single device (i.e., the AMD Radeon R9 295X2 graphics card) at about $1,500. Moreover, large clusters are adopting the use of these relatively inexpensive and powerful devices as a way of accelerating parts of the applications they are running. Presently, most of the top 10 systems from the TOP500 list (November 2013) are equipped with accelerators, and in particular Titan, the second system in the list, achieved 17.59 PetaFlop on the Linpack benchmark also thanks to its 18,688 Nvidia Tesla K20 devices.

Driven by the demand of the game industry, GPUs have completed a steady transition from mainframes to workstations PC cards, where they emerge nowadays like a solid and compelling alternative to traditional computing platforms. GPUs deliver extremely high floating-point performance and massively parallelism at a very low cost, thus promoting a new concept of the high performance computing market. For example, in heterogeneous computing, processors with different characteristics work together to enhance the application performance taking care of the power budget. This fact has attracted many researchers and encouraged the use of GPUs in a broader range of applications, particularly in the field of bioinformatics. Developers are required to leverage this new landscape of computation with new programming models, which ease the developers task of writing programs to run efficiently on such platforms altogether [50].

The most popular microprocessor companies such as NVIDIA, ATI/AMD, or Intel, have developed hardware products aimed specifically at the heterogeneous or massively parallel computing market: Tesla products are from NVIDIA, Fire-stream is the AMDs device line, and Intel Xeon Phi comes from Intel. They have also released software components, which provide simpler access to this computing power. CUDA (Compute Unified Device Architecture) is NVIDIAs solution as a simple block-based API for programming; AMDs alternative was called Stream Computing, while Intel relies directly on X86-based programming. OpenCL [51] emerged as an attempt to unify all of these models with a superset of features, being the best broadly supported multiplatform data parallel programming interface for heterogeneous computing, including GPUs, accelerators, and similar devices.

Although these efforts in developing programming models have made great contributions to leverage the capabilities of these platforms, developers have to deal with a massively parallel and high-throughput-oriented architecture [52], which is quite different than traditional computing architectures. Moreover, GPUs are being connected with CPUs through PCI Express bus to build heterogeneous parallel computers, presenting multiple independent memory spaces, a wide spectrum of high speed processing functions, and some communication latencies between them. These issues drastically increase scaling to a GPU cluster, bringing additional sources of complexity and latency. Therefore, programmability on these platforms is still a challenge, and thus many research efforts have provided abstraction layers avoiding dealing with the hardware particularities of these accelerators and also extracting transparently high level of performance, providing portability across operating systems, host CPUs, and accelerators. For example, libraries and interfaces exist for developing with popular programming languages like OMPSs for OpenMP or OpenACC API, which describes a collection of compiler directives to loops specific regions of code in standard programming languages such as C, C++, or Fortran. Although the complexity of these architectures is high, the performance that such devices are able to provide justifies the great interest and efforts in porting bioinformatics application on them [53].

### 3.3. Xeon Phi

Based on Intel's Many Integrated Core (MIC) x86-based architecture, Intel Xeon Phi coprocessors provide up to 61 cores and 1.2 Teraflops of performance. These devices equip the first supercomputer of the TOP500 list (November 2013), Tianhe-2. In terms of usability, there are two ways an application can use an Intel Xeon Phi: in offload mode or in native mode. In offload mode the main application is running on the host, and it only offloads selected (highly parallel, computationally intensive) work to the coprocessor. In native mode the application runs independently, on the Xeon Phi only, and can communicate with the main processor or other coprocessors through the system bus.

The performance of these devices heavily depends on how well the application fits the parallelization paradigm of the XeonPhi and in relation to the optimizations that are performed. In fact, since the processors on the Xeon Phi have a lower clock frequency with respect to the common Intel processor unit (such as for example the Sandy Bridge), applications that have long sequential part of the algorithm are absolutely not suitable for the native mode. On the other hand, even if the programming paradigm of these devices is standard C/C++, which makes their use simpler with respect to the necessity of exploiting a different programming language such as CUDA, in order to achieve good performance, the code must be heavily optimized to fit the characteristics of the coprocessor (i.e., exploiting optimizations introduced by the Intel compiler and the MKL library).

Looking at the performance tests released by Intel [54], the baseline improvement of supporting two Intel Sandy Bridge by offloading the heavy parallel computational to an Intel Xeon Phi gives an average improvement of 1.5 in the scalability of the application that can reach up to 4.5 of gain after a strong optimization of the code. For example, considering typical tools for bioinformatics sequence analysis: BWA (Burrows-Wheeler Alignment) [55] reached a baseline improvement of 1.86 and HMMER of 1.56 [56]. With a basic recompilation of Blastn for the Intel Xeon Phi [57] there is an improvement of 1.3, which reaches 4.5 after some modifications to the code in order to improve the parallelization approach. Same scalability figures for Abyss, which scales 1.24 with a basic porting and 4.2 with optimizations in the distribution of the computational load. Really good performance is achieved for Bowtie, which improves the code passing from a scalability of 1.3 to 18.4.

Clearly, the real competitors of the Intel Xeon Phi are the GPUs devices. At the moment, the comparison between the best devices provided by Intel—Xeon Phi 7100—and Nvidia—K40—shows that the GPU is on average 30% more performing [58], but the situation can vary in the future.

### 3.4. Cloud Computing

Advances in life sciences and information technology bring profound influences on bioinformatics due to its interdisciplinary nature. For this reason, bioinformatics is experiencing a new trend in the way analysis is performed: computation is moving from in-house computing infrastructure to cloud computing delivered over the Internet. This has been necessary in order to handle the vast quantities of biological data generated by high-throughput experimental technologies. Cloud computing in particular promises to address Big Data storage and analysis issues in many fields of bioinformatics. But moving data to the cloud can also be a problem; so hybrid solutions of cloud computing are arising. The point can be summarized as follows: data that is too big to be processed conventionally is also too big to be transported anywhere. IT is undergoing an inversion of priorities: the code to perform the analysis has to be moved, not the data. Virtualization represents an enabling technology to achieve this result.

One of the most famous free portal/software for the analysis of bioinformatics data, Galaxy by the Penn state University, is available on cloud [59]. The idea is that with sporadic availability of data, individuals and labs may have a need to, over a period of time, process greatly variable amounts of data. Such variability in data volume imposes variable requirements on availability of compute resources used to process given data. Rather than having to purchase and maintain desired compute resources or having to wait a long time for data processing jobs to complete, the Galaxy Team has enabled Galaxy to be instantiated on cloud computing infrastructures, primarily Amazon Elastic Compute Cloud (EC2). An instance of Galaxy on the cloud behaves just like a local instance of Galaxy except that it offers the benefits of cloud computing resource availability and pay-as-you-go resource ownership model. Having simple access to Galaxy on the cloud enables as many instances of Galaxy to be acquired and started as is needed to process given data. Once the need subsides, those instances can be released as simply as they were acquired. With such a paradigm, one pays only for the resources they need and use, while all the other concerns and costs are eliminated.

For a complete review on bioinformatics clouds for Big Data manipulation, see [60]. Concerning the exploitation of cloud computing to cope with the data flow produced by high-throughput molecular biology technologies, see [61].

## 4. Semantics, Ontologies, and Open Format for Data Integration

The previous sections focused mainly on how to analyse Big Data for inferring correlations, but the extraction of actual new knowledge requires something more. The key challenges in making use of Big Data lie, in fact, in finding ways of dealing with heterogeneity, diversity, and complexity of the information, while its volume and velocity hamper solutions are available for smaller datasets such as manual curation or data warehousing.

Semantic web technologies are meant to deal with these issues. The development of metadata for biological information on the basis of semantic web standards can be seen as a promising approach for a semantic-based integration of biological information [62]. On the other hand, ontologies, as formal models for representing information with explicitly defined concepts and relationships between them, can be exploited to address the issue of heterogeneity in data sources.

In domains like bioinformatics and biomedicine, the rapid development and adoption of ontologies [63] prompted the research community to leverage them for the integration of data and information. Finally, since the advent of linked data a few years ago, it has become an important technology for semantic and ontologies research and development. We can easily understand linked data as being a part of the greater Big Data landscape, as many of the challenges are the same. The linking component of linked data, however, puts an additional focus on the integration and conflation of data across multiple sources.

### 4.1. Semantics

The semantic web is a collaborative movement, which promoted standard for the annotation and integration of data. By encouraging the inclusion of semantic content in data accessible through the Internet, the aim is to convert the current web, dominated by unstructured and semistructured documents, into a web of data. It involves publishing information in languages specifically designed for data: Resource Description Framework (RDF), Web Ontology Language (OWL), SPARQL (which is a protocol and query language for semantic web data sources), and Extensible Markup Language (XML) [64].

RDF represents data using subject-predicate-object triples, also known as “statements.” This triple representation connects data in a flexible piece-by-piece and link-by-link fashion that forms a directed labelled graph. The components of each RDF statement can be identified using Uniform Resource Identifiers (URIs). Alternatively, they can be referenced via links to RDF Schemas (RDFS), Web Ontology Language (OWL), or to other (nonschema) RDF documents. In particular, OWL is a family of knowledge representation languages for authoring ontologies or knowledge bases. The languages are characterised by formal semantics and RDF/XML-based serializations for the semantic web. In the field of biomedicine, a notable example is the Open Biomedical Ontologies (OBO) project, which is an effort to create controlled vocabularies for shared use across different biological and medical domains. OBO belongs to the resources of the U.S. National Center for Biomedical Ontology (NCBO), where it will form a central element of the NCBO's BioPortal. The interrogation of these resources can be performed using SPARQL, which is an RDF query language similar to SQL, for the interrogations of databases, able to retrieve and manipulate data stored in RDF format. For example, BioGateway [65] organizes the SwissProt database, along with Gene Ontology Annotations (GOA), into an integrated RDF database that can be accessed through a SPARQL query endpoint, allowing searches for proteins based on a combination of GO and SwissProt data.

The support for RDF in high-throughput bioinformatics applications is still small, although researchers can already download the UniProtKB and its taxonomy information using this format or they can get ontologies in OWL format, such as GO [66]. The biggest impact RDF and OWL can have in bioinformatics, though, is to help integrate all data formats and standardise existing ontologies. If unique identifiers are converted to URI references, ontologies can be expressed in OWL, and data can be annotated via these RDF-based resources. The integration between them is a matter of merging and aligning the ontologies (in case of OWL using the “rdf:sameAs” statement). After the data has been integrated we can use the plus that comes with RDF for reasoning: context embeddedness. Organizations in the life sciences are currently using RDF for drug target assessment [67, 68], and for the aggregation of genomic data [69]. In addition, semantic web technologies are being used to develop well-defined and rich biomedical ontologies for assisting data annotation and search [70, 71], the integration of rules to specify and implement bioinformatics workflows [72], and the automation for discovering and composing bioinformatics web services [73].

### 4.2. Ontologies

An ontology layer is often an invaluable solution to support data integration [74], particularly because it enables the mapping of relations among data stored in a database. Belonging to the field of knowledge representation, an ontology is a collection of terms within a particular domain organised in a hierarchical structure that allows searching at various levels of specificity. Ontologies provide a formal representation of a set of concepts through the description of individuals, which are the basic objects, classes, that are the categories which objects belong to, attributes, which are the features the objects can present, and relations, that are the ways objects can be related to one another. Due to this “tree” (or, in some cases, “graph”) representation, ontologies allow the link of terms from the same domain, even if they belong to different sources in data integration contexts, and the efficient matching of apparently diverse and distant entities. The latter aspect can not only improve data integration, but even simplify the information searching.

In the biomedical context a common problem concerns, for example, the generality of the term cancer [75]. A direct query on that term will retrieve just the specific word in all the occurrences found into the screened resource. Employing a specialised ontology (i.e., the human disease ontology—DOID) [76] the output will be richer, including terms such as sarcoma and carcinoma that will not be retrieved otherwise. Ontology-based data integration involves the use of ontologies to effectively combine data or information from multiple heterogeneous sources [63]. The effectiveness of ontology-based data integration is closely tied to the consistency and expressivity of the ontology used in the integration process. Many resources exist that have ontology support: SNPranker [77], G2SBC [78], NSD [79], TMARepDB [80], Surface [81], and Cell cycle DB [82].

A useful instrument for ontology exploration has been developed by the European Bioinformatics Institute (EBI), which allows easily visualising and browsing ontologies in the OBO format: the open source Ontology Lookup Service (OLS) [83]. The system provides a user-friendly web-based single point to look into the ontologies for a single specific term that can be queried using a useful autocompletion search engine. Otherwise it is possible to browse the complete ontology tree using an Ajax library, querying the system through a standard SOAP web service described by a WSDL descriptor.

The following ontologies are commonly used for annotation and integration of data in the biomedical and bioinformatics.

- Gene ontology (GO) [84], which is the most exploited multilevel ontology in the biomolecular domain. It collects genome and proteome related information in a graph-based hierarchical structure suitable for annotating and characterising genes and proteins with respect to the molecular function (i.e., GO:0070402: NADPH binding) and biological process they are involved in (i.e., GO:0055114: oxidation reduction), and the spatial localisation they present within a cell (i.e., GO:0043226: organelle).
- KEGG ontology (KOnt), which provides a pathway based annotation of the genes in all organisms. No OBO version of this ontology was found, since it has been generated directly starting from data available in the related resource [85].
- Brenda Tissue Ontology (BTO) [86], to support the description of human tissues.
- Cell Ontology (CL) [87], to provide an exhaustive organisation about cell types.
- Disease ontology (DOID) [76], which focus on the classification of breast cancer pathology compared to the other human diseases.
- Protein Ontology (PRO) [88], which describes the protein evolutionary classes to delineate the multiple protein forms of a gene locus.
- Medical Subject Headings thesaurus (MESH) [89], which is a hierarchical controlled vocabulary able to index biomedical and health-related information.
- Protein structure classification (CATH) [90], which is a structured vocabulary used for the classification of protein structures.

### 4.3. Linked Data

Linked data describes a method for publishing structured data so that they can be interlinked, making clearer the possible interdependencies. This technology is built upon the semantic web technologies previously described (in particular it uses HTTP, RDF, and URIs), but rather than using them to serve web pages for human readers, it extends them to share information in a way that can be read automatically by IT systems.

The linked data paradigm is one approach to cope with Big Data as it advances the hypertext principle from a web of documents to a web of rich data. The idea is that after making data available on the web (in an open format and with an open license) and structuring them in a machine-readable fashion (e.g., Excel instead of image scan of a table), researchers must work to annotate this information with open standards from W3C (RDF and SPARQL), so that people can link their own data to other people's data to provide context information.

In the field of bioinformatics, a first attempt to publish linked data has been performed by the Bio2RDF project [91]. The project's goal is to create a network of coherently linked data across the biological databases. As part of the Bio2RDF project, an integrated bioinformatics warehouse on the semantic web has been built. Bio2RDF has created a RDF warehouse that serves over 70 million triples describing the human and mouse genomes [92].

A very important step towards the use of linked data in the computational biology field has been done by the abovementioned EBI [93], which developed an infrastructure to access its information by exploiting this paradigm. In detail, the EBI RDF platform allows explicit links to be made between datasets using shared semantics from standard ontologies and vocabularies, facilitating a greater degree of data integration. SPARQL provides a standard query language for querying RDF data. Data that have been annotated using ontologies, such as DOID and the GO, can be integrated with other community datasets, providing the semantics support to perform rich queries. Publishing such datasets as RDF, along with their ontologies, provides both the syntactic and semantic integration of data, long promised by semantic web technologies. As the trend toward publishing life science data in RDF increases, we anticipate a rise in the number of applications consuming such data. This is evident in efforts such as the Open PHACTS platform [94] and the AtlasRDF-R package [95]. The final aim is that EBI RDF platform can enable such applications to be built by releasing production quality services with semantically described RDF, enabling real biomedical use cases to be addressed.

## 5. Data Access and Security

Besides improving the search capabilities via ontologies, metadata, and linked data for accessing data efficiently, the usability aspect is also a fundamental topic for Big Data. Scientists want to focus on their specific research while creating and analysing data without the need to know all the low-level burdens related to the underlying data management infrastructures. This demand can be addressed by science gateways that are single points of entry to applications and data across organizational boundaries. Data security is another aspect that must be addressed when providing access to Big Data, in particular while working in the healthcare sector.

### 5.1. Science Gateways

The overall goal of science gateways is to hide the complex underlying infrastructure and to offer intuitive user interfaces for job, data, and workflow management. In the last decade diverse mature frameworks, libraries, and APIs have been evolved, which allow the enhanced development of science gateways. While distributed job and workflow management are widely supported in frameworks like EnginFrame [96], implemented on top of the standards-based portal framework Liferay [97] or the proprietary workflow-enabled science gateway Galaxy [98–100], the data management is often not completely satisfactory. Also content management systems such as Drupal [101] and Joomla [102] or the high-level framework Django [103] still lack the support of sophisticated data management features for data on a large scale.

VectorBase [104], for example, is a mature science gateway for invertebrate vectors of human pathogens developed in Drupal offering large sets of data and tools for further analysis. While this science gateway is widely used with a user community of over 100,000 users, the data management is directly dependent on the underlying file system, without additional data management features tailored to Big Data. The sophisticated metadata management in VectorBase has been developed by the VectorBase team from scratch since Drupal lacks metadata management capabilities.

The requirement for processing Big Data in a science gateway is reflected in a few current developments in the context of standardized APIs and frameworks. Agave [105] provides powerful API for developing intuitive user interfaces for distributed job management, which has been extended in a first prototype with metadata management capabilities, and allows the integration with distributed file systems. Globus Transfer [106] forms a data bridge, which supports the storage protocol GridFTP [107]. It offers a web-based user interface and community features similar to DropBox [108]. The users are enabled to easily share data and manage it in distributed environments. The Biomedical Research Informatics Network (BIRN) [109], for example, is a national initiative to advance biomedical research via data sharing and collaborations and the corresponding infrastructure applies Globus Transfer for moving large datasets. Data Avenue [110] follows an analogous concept as Globus Transfer and provides a web-based user interface as well. Additionally, it will be integrated in the workflow-enabled science gateway WS-PGRADE [111], which is the flexible user interface of gUSE. The extension by Data Avenue enhances the data management in the science gateway framework significantly so that not only jobs and workflows can be distributed to diverse grid, cloud, cluster, and collaborative infrastructures, but also distributed data can be efficiently managed via storage protocols like HTTP(s), Secure FTP (SFTP), GridFTP, Storage Resource Management (SRM) [112], Amazon Simple Storage Service (S3) [113], and integrated Rule-Oriented Data System (iRODS) [114].

An example of a science gateway in the bioinformatics filed is MoSGrid (molecular simulation grid) [115] that supports the molecular simulation community with an intuitive user interface in the areas of quantum chemistry, molecular dynamics, and docking screening. It has been developed on the top of WS-PGRADE/gUSE and features metadata management with search capabilities via Lucene [116] and distributed data management via the object-based distributed file system XtreemFS [117]. While these capabilities have been developed before Data Avenue was available in WS-PGRADE, the layered architecture of the MoSGrid science gateways has been designed to allow the integration of further data management systems and thus can be extended for Data Avenue.

### 5.2. Security

Whatever technology is used, the distributed data management for biomedical applications with community options for sharing data requires especially secure authentication and secure measures to assure strict access policies and the integrity of data [118]. Medical bioinformatics, in fact, is often concerned with sensitive and expensive data such as projects contributing to computer-aided drug design or in environments like hospitals. The distribution of data increases the complexity and involves data transfer through many network devices. Thus, data loss or corruption can occur.

GSI (Grid Security Infrastructure) [119] has been designed for the authentication via X.509 certificates and assures the secure access to data. iRODS and XtreemFS, for example, support GSI for authentication. Both use enhanced replication mechanisms to warrant the integrity of data including the prevention of loss. Amazon S3 follows a username and password approach while owner of an instance can grant access to the data via ACLs (access control lists). The corresponding Amazon web services also replicate the data over diverse instances and examine MD5 checksums to check whether the data transfer was fully successful and the transferred files unchanged. The security mechanisms in Data Avenue as well as in Globus Transfer are based on GSI. Globus Transfer applies Globus Nexus [105] as security platform, which is capable of creating a full identity management with authentication and group mechanisms. Globus Nexus can serve as security service layer for distributed data management systems and can connect to diverse storages like Amazon S3.

## 6. Perspectives and Open Problems

Data is considered the Fourth Paradigm in science [120], besides experimental, theoretical, and computational sciences. This is becoming particularly true in computational biology, where, for example, the approach “sequence first, think later” is rapidly overcoming the hypothesis-driven approach. In this context, Big Data integration is really critical for bioinformatics that is “the glue that holds biomedical research together.”

There are many open issues for Big Data management and analysis, in particular in the computational biology and healthcare fields. Some characteristics and open issues of these challenges have been discussed in this paper, such as architectural aspects and the capability of being flexible enough to collect and analyse different kind of information. It is critical to face the variety of the information that should be managed by such infrastructures, which should be organized in scheme-less contexts, combining both relaxed consistency and a huge capacity to digest data. Therefore, a critical point is that relational databases are not suitable for Big Data problems. They lack horizontal scalability, need hard consistency, and become very complex when there is the need to represent structured relationships. Nonrelational databases (NoSQL) are the interesting alternative to data storage because they combine the scalability and flexibility.

From the computational point of view, the novel idea is that jobs are directly responsible of managing input data, through suitable procedures for partitioning, organizing, and merging intermediate results. Novel algorithm will contain large parts of not functional code, but essential for exploiting housekeeping tasks. Due to the practical impossibility of moving all the data across geographical dispersed sites, there is the need of computational infrastructure able to combine large storage facilities and HPC. Virtualization can be the key in this challenge, since it can be exploited to achieve storage facilities able to leverage in-memory key/value databases to accelerate data-intensive tasks.

The most important initiatives for the usage of Big Data techniques in medical bioinformatics are related to scientific research efforts, as described in the paper. Nevertheless, some commercial initiatives are available to cope with the huge quantity of data produced nowadays in the field of molecular biology exploiting high-throughput omics technologies for real-life problems. These solutions are designed to support researchers working in computational biology mainly using Cloud infrastructures. Examples are Era7 Bioinformatics [121], DNAnexus [122], Seven Bridge Genomics [123], EagleGenomics [124], and MaverixBio [125]. Noteworthy, also large providers of molecular biology instrumentations, such as Illumina [126], and huge service providers, such as BGI [127], have Cloud-based services to support their customers.

Hospitals are also considering to hiring Big Data solutions in order to provide “support services for researchers who need assistance with managing and analyzing large medical data sets” [128]. In particular, McKinsey & Company stated already in April 2013 that Big Data will be able to revolutionize pharmaceutical research and development within clinical environments, by targeting the diverse user roles physicians, consumers, insurers, and regulators [129]. In 2014 they predicted that Big Data could lead to a reduction of research and development costs for the pharmaceutical industry by approximately 35% (about $40 billion) [130]. Drugmakers, healthcare providers, and health analysis companies are collaborating on this topic; for example, drugmaker GlaxoSmithKline PLC and the health analysis company SAS Institute Inc. work on a private Cloud for pharmaceutical industry sharing securely anonymized data [130]. Open data especially can be exploited by patient communities such as PatientsLikeMe.com [131] containing invaluable information for further data mining.

It is therefore clear that in the Big Data field there is much to do in terms of making these technologies efficient and easy-to-use, especially considering that even small and medium-size laboratories are going to use them in a close future.
